# Network Pharmacology and Bioinformatics Methods Reveal the Mechanism of Zao-Jiao-Ci in the Treatment of LSCC

**DOI:** 10.1155/2021/8862821

**Published:** 2021-06-25

**Authors:** Feng Xiang, Linman Li, Jieling Lin, Shasha Li, Guiyuan Peng

**Affiliations:** ^1^Department of Otolaryngology, YangZhou Hospital of Traditional Chinese Medicine, JiangSu, YangZhou, China; ^2^The Second Clinical College, Guangzhou University of Chinese Medicine, Guangzhou, Guangdong, China; ^3^Department of Otolaryngology, Guangdong Province Hospital of Chinese Medicine, Guangzhou, Guangdong, China

## Abstract

**Objective:**

Zao-Jiao-Ci (ZJC), a traditional Chinese medicine, is considered as a promising candidate to treat laryngeal squamous cell carcinoma (LSCC). However, the underlying molecular mechanism remains unclear.

**Methods:**

Gene expression profiles of GSE36668 were available from the GEO database, and differentially expressed genes (DEGs) of LSCC were obtained by *R* package; subsequently, enrichment analysis on KEGG and GO of DEGs was performed. The active ingredients of ZJC were screened from the TCMSP database, and the matched candidate targets were obtained by PharmMapper. Furthermore, we constructed protein-protein interaction (PPI) networks of DEGs and candidate targets, respectively, and we screened the core network from the merged network through combining the two PPI networks using Cytoscape 3.7.2. The key targets derived from the core network were analyzed to find out the associated KEGG signal enrichment pathway. By the GEPIA online website, Kaplan–Meier analysis was used to complete the overall survival and disease-free survival of the selected genes in the core module.

**Results:**

We identified 96 candidate targets of ZJC and 86 DEGs of LSCC, the latter including 50 upregulated genes and 36 downregulated genes. DEGs were obviously enriched in the following biological functions: extracellular structure organization, the extracellular matrix organization, and endodermal cell differentiation. The 60 key targets from the core network were enriched in the signal pathways including transcriptional misregulation cancer, cell cycle, and so on. We found that LSCC patients with high expression of HIST1H3J, HIST1H3F, and ITGA4 had worse overall survival, while higher expression of NTRK1, COPS5, HIST1H3A, and HIST1H3G had significantly worse disease-free survival.

**Conclusion:**

It suggested that the interaction between ZJC and LSCC was related to the signal pathways of transcriptional misregulation cancer and cell cycle, revealing that it may be the mechanism of ZJC in the treatment of LSCC.

## 1. Introduction

Laryngeal squamous cell carcinoma (LSCC) is the most common malignancy of the larynx, and its clinical manifestations are hoarseness, stridor, dyspnea, and even dysphagia [1, 2]. Disappointingly, despite various technologies such as surgery, laser therapy, and chemoradiation have advanced recently, and the survival rate has not improved because of a high rate of recurrence and metastasis [3, 4]. Therefore, in order to improve survival rates of the patients, there is an urgent need for effective treatment.

An increasing number of studies confirmed that traditional Chinese medicine (TCM) including multiple ingredients and targets play a critical role in the treatment of cancer. Zao-Jiao-Ci (ZJC), also known as *Gleditsia sinensis*, is a traditional Chinese medicine with a variety of bioactivities, especially antitumor activity, which has been widely used in clinic [5]. It was investigated that the ethanol extract of *Gleditsia sinensis* (EEGS) could suppress the growth of human colon cancer HCT116 cells in vitro and in vivo [6]. The extract of *Gleditsia sinensis* fruit performed inhibitory effects on esophageal squamous cell carcinoma (ESCC) cells, breast cancer MCF-7 cells, hepatoblastoma HepG2 cells, and so on [7, 8]. However, there is no study to investigate the anticancer effect of *Gleditsia sinensis* on LSCC, and the mechanism remains unclear.

Network pharmacology has exhibited specific utility in analyzing multicomponent and multitarget, consistent with the therapy hypothesis of complex diseases. By constructing a multilevel, multifaceted network model comprised of components, targets, pathways, and diseases, we can investigate TCM in the treatment of disease involved in the regulation of a variety of signaling pathways, key targets taxa, and biological process analysis, aiming to reveal the mechanism from the molecular level [9].

In this study, we used network pharmacology to investigate whether ZJC exerts anticancer effects on LSCC based on the GEO microarray dataset. And through the pathway enrichment analysis of the interaction targets between differentially expressed gene (DEGs) of LSCC and key node targets of ZJC, we further predicted the therapeutic mechanism of ZJC on LSCC. To our knowledge, this study is the first to explore the efficacy and mechanism of ZJC on LSCC, providing theoretical support and directions for further basic research.

## 2. Methods

### 2.1. Active Ingredients Screening and Targets Prediction for ZJC

Through the Traditional Chinese Medicine Systems Pharmacology Database and Analysis Platform (TCMSP), all components of ZJC could be found by searching the term “Zao-Jiao-Ci.” We set oral bioavailability (OB) > 30% and drug-likeness (DL) > 0.18 as screening conditions supported by the published literatures to obtain the final active ingredients [10, 11]. PharmMapper server is the first webserver for potential drug targets identification through large-scale reverse pharmacophore mapping strategy [12]. The MOL structure of active ingredients provided by TCMSP was input into PharmMapper server (http://lilab.ecust.edu.cn/pharmmapper/) to get the targets of the pharmacophore model. The first 15 targets sorted by the fit score were seemed as candidate targets of ZJC.

### 2.2. Active Ingredient-Target PPI Network Construction

To explore the association between ingredients and targets, we established an interaction network. Cytoscape 3.7.2, one of the most favorite open-source software tools, provides visually biomedical interaction networks composed of protein, gene, and other types of interactions. It was used to develop an active ingredient-target PPI network to visualize the relationship between the active ingredients and their targets of ZJC.

### 2.3. GEO Data Collection and DEGs Identification

The original data series GSE84957 was downloaded from the Gene Expression Omnibus (GEO) microarray dataset, which contained gene expression profiles of 18 tissue samples (9 LSCC tumor tissues and 9 normal tissues). The *R* language was used to process the original data sets, and the RMA algorithm of Affy software package was used to perform background correction and quartile standardization of the expression matrix. The gene ID, the gene probe name of the expression matrix, was replaced by the gene symbol provided by the GPL17843 Agilent-042818 Human lncRNA Microarray 8_24_v2 platform, and the average value of multiple probes for the same gene was used for analysis. Limma package was used to identify the significant differentially expressed genes (DEGs) according to *P* < 0.01, |log2 (FC)| > 3.

The screened DEGs were mapped into a volcano map using the *R* language heatmap package for intuitive vision; finally, the clusterProfiler package was used to carry out GO enrichment analysis and KEGG pathway enrichment analysis for DEGs.

### 2.4. PPI Network Construction

BisoGenet plugin, comprising of six available PPI databases (the Biological General Repository for Interaction Datasets (BioGRID), Biomolecular Interaction Network Database (BIND), Molecular Interaction Database (MINT), Human Protein Reference Database (HPRD), and Database of Interacting Proteins (DIP)), was used to build the PPI network for DEGs and candidate target genes, respectively [13]. Then, the merged network was conducted for the two PPI networks. We filtered the output nodes with degrees of freedom greater than 2 times the median of all nodes according to the indicators of degree and betweenness centrality. Then, a core PPI network was constructed using CytoNCA, a Cytoscape plugin. The ClueGO plugin was used for the KEGG signaling pathway enrichment analysis. *P* < 0.01 was taken as the inclusion standard for pathway items. The results of enrichment analysis were presented in the form of the pie chart and nodes.

### 2.5. Cluster of the Core PPI Network

The MCODE plugin in Cytoscape software was used to screen the highly clustered important modules in the core PPI network. We set the parameters as degree cutoff = 2 and *κ*-core = 2 and conducted KEGG signaling pathway enrichment analysis for the most significantly clustered modules.

### 2.6. Gene Expression Data of the Core Cluster for LSCC

The correlation between survival rates of LSCC patients (disease-free survival rate and overall survival rate) and the gene expression levels (NTRK1, COPS5, HIST1H3A, HIST1H3G, HIST1H3J, HIST1H3J, HIST1H3F, and ITGA4) were calculated using GEPIA online database (http://GEPIA.cancer pku.cn/) [14].

## 3. Results

### 3.1. Active Ingredients and Targets of ZJC

Searching for all the reported components in the TCMSP database, 30 active ingredients of ZJC were collected. Sequentially, only 11 active ingredients were retained which conformed to OB > 30% and DL > 0.18, such as fisetin, fustin, flavanone, and kaempferol (Table 1). Then, 96 candidate targets of the above 11 active components were obtained after the duplicate targets were excluded.

### 3.2. Active Ingredients-Targets PPI Network Construction

**Figure 1:**
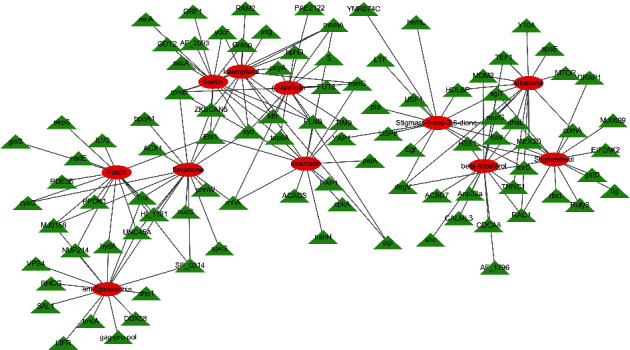
Active ingredients-targets network of ZJC. The blue circles represent the active ingredients, the red diamonds represent the targets, and the interaction between the two is represented by gray edges.

A PPI network of the active components and relevant targets, containing 107 nodes and 165 edges, was constructed by the network graphing tool Cytoscape 3.7.2. The 11 active ingredients could be connected with multiple targets, respectively, and each target also could be connected with multiple active ingredients, which directly demonstrated the relationship between active ingredients and targets of ZJC (Figure 1).

### 3.3. LSCC Differentially Expressed Genes (DEGs)

**Figure 2:**
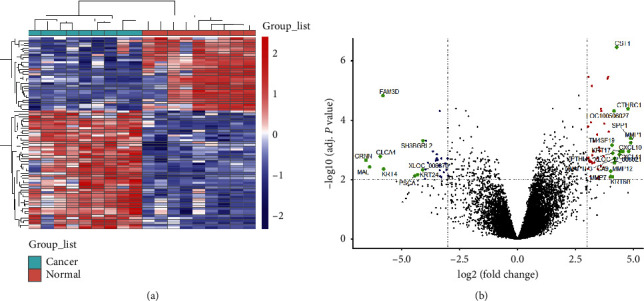
Volcano plot of gene expression and heatmap of DEGs. (a) The rows represent genes and the columns represent samples. The first 9 columns are tumor samples and the last 9 columns are normal samples. Red represents high gene expression, and blue represents low gene expression. (b) The red, blue, and green dots represent the differentially expressed genes between the tumor tissues and normal tissues of LSCC, among which the red represents the upregulated genes in the tumor tissues, the blue represents the downregulated genes, and the green represents the genes of logFC > 4, while the black represents the insignificantly different genes.

By analyzing the gene chip of GSE84957, a total of 81 genes with significant different expression of the LSCC tissues compared with adjacent nonneoplastic tissues were obtained, among which 50 genes were upregulated and 31 genes were downregulated in tumor tissues (Table 2; Figure 2).

### 3.4. GO Enrichment Analysis and KEGG Pathway Analysis for DEGs

**Figure 3:**
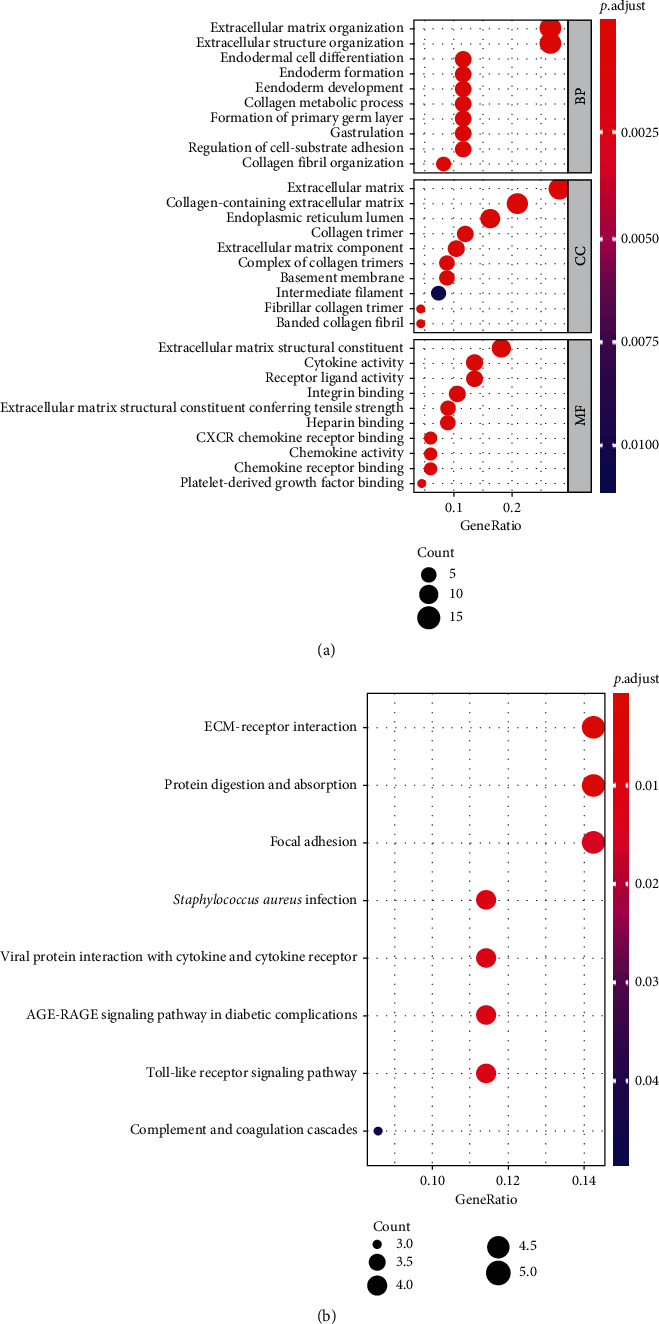
GO enrichment analysis and KEGG pathways analysis on DEGs. (a) GO enrichment analysis: the top 10 terms of biological process, cellular component, and molecular function with *P* < 0.05. (b) KEGG pathways analysis: the top 3 terms of KEGG enrichment pathway with *P* < 0.05.

GO enrichment analysis was used to explore the molecular mechanism of DEGs. The results were given as follows: (i) in the BP category, DEGs were mostly enriched in the extracellular structure organization, the extracellular matrix organization, endodermal cell differentiation, endoderm formation, and endoderm development; (ii) in the category of CC, DEGs were mainly enriched in the extracellular matrix, collagen-containing extracellular matrix, endoplasmic reticulum lumen, collagen trimer, and extracellular matrix component; (iii) in the MF category, extracellular matrix structural constituent, cytokine activity, and receptor ligand activity were selected for main MF. The results of KEGG pathway analysis showed that ECM-receptor interaction, protein digestion and absorption, focal adhesion, *Staphylococcus aureus* infection, and viral protein interaction with cytokine and cytokine receptor were the major pathways involved in DEGs (Figure 3).

### 3.5. PPI Network Construction and Key Targets Screening

**Figure 4:**
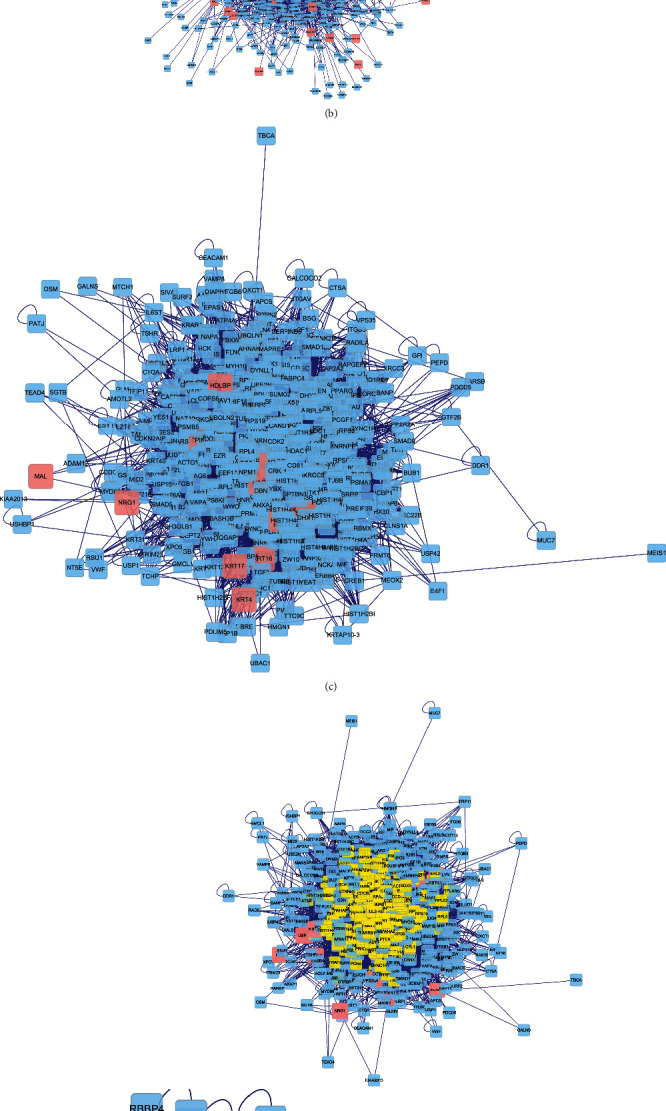
Identification of key targets for ZJC against LSCC. (a) PPI of ZJC targets. (b) PPI of DEGs in LSCC. (c) The intersections of the two PPI networks of ZJC and DEGs. (d) Topological screening of the interactive PPI network based on degree and betweenness centrality. The same type of the signaling pathway is represented by nodes of the same color, and the size of the node represents the significance of the signaling pathway. The higher the significance of the signaling pathway is, the larger the node is, indicating that the importance of the pathway is higher.

A PPI network based on the targets of ZJC active ingredients was constructed. It showed that ZJC had direct or indirect correlation with the 1572 targets, and there were 29,098 interconnections between these targets. At the same time, the PPI network was mapped for DEGs, and 2,262 targets were directly or indirectly related to LSCC, with 50,181 interconnections between these targets. Then, the intersections of the two PPI networks were used to construct a merged network with 510 nodes and 10950 edges (Figures 4(a)–4(c)). Furthermore, we analyzed the topological properties of the nodes in the merged network of the protein interactions to find the key nodes. Finally, 60 key nodes were identified through the network topology analysis (Figure 4(d) and Table 3).

### 3.6. KEGG Pathway Analysis and Main Module of the Core PPI Network

**Figure 5:**
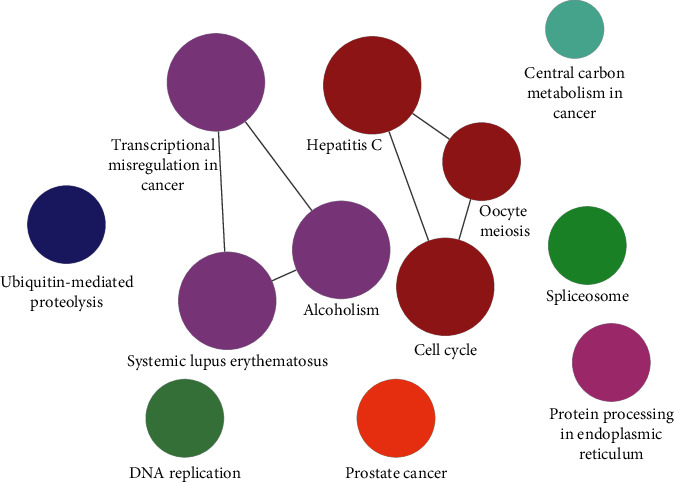
Vital terms of KEGG enrichment analysis of the key targets.

**Figure 6:**
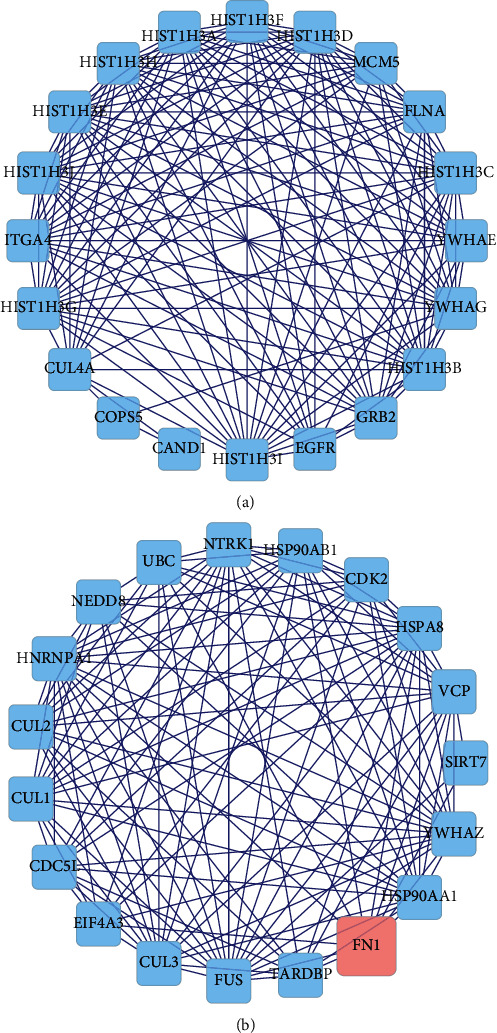
The two main modules of the core PPI network of ZJC against LSCC.

The KEGG signaling pathways analysis suggested that 60 key targets were mainly enriched in cell cycle, central carbon metabolism in cancer, and DNA replication, indicating the mechanisms of ZJC in the treatment of LSCC. The other signaling pathways included prostate cancer, protein processing in endoplasmic reticulum, spliceosome, transcriptional misregulation in cancer, and ubiquitin-mediated proteolysis (Figure 5). Through the MCODE plugin, two main modules of the core PPI network were obtained, one of which was functionally enriched in alcoholism, transcriptional misregulation in cancer, and systemic lupus erythematosus (Figure 6).

### 3.7. LSCC Survival Analysis

**Figure 7:**
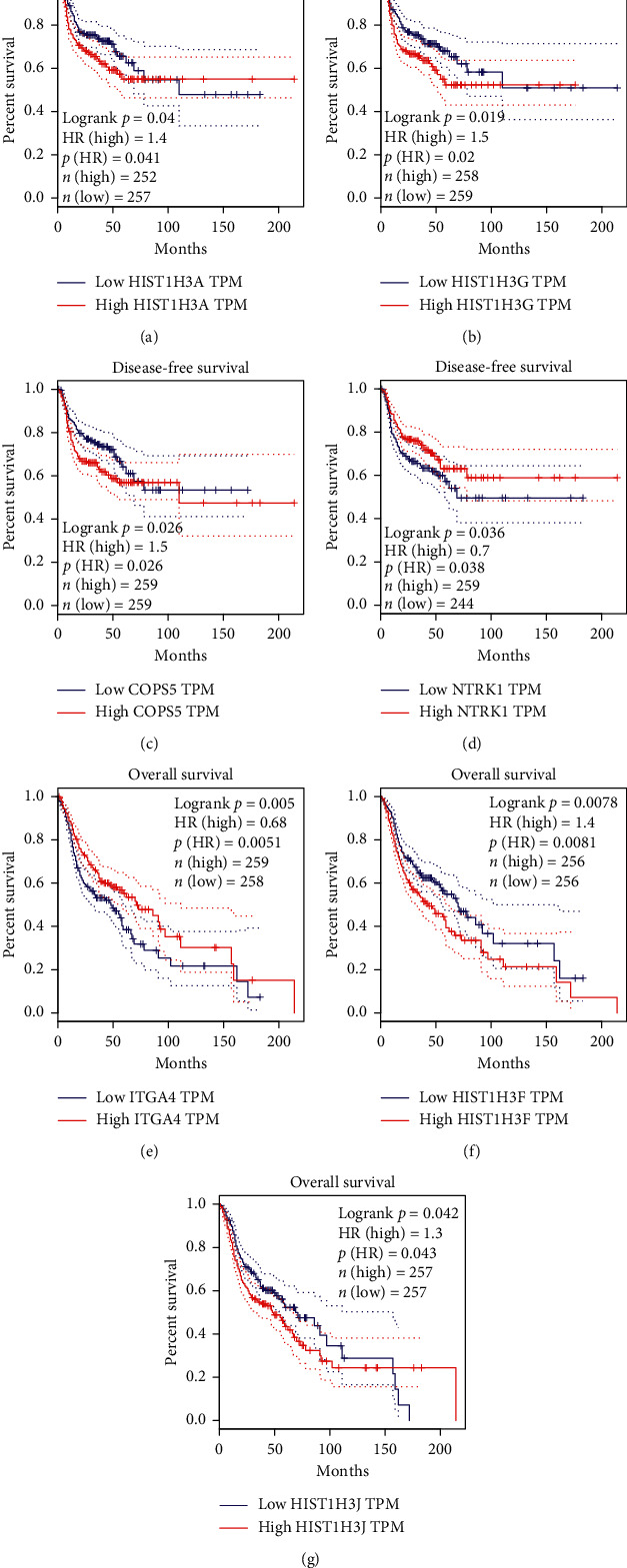
Disease-free survival analyses and overall survival analyses of LSCC. (a)–(d) Disease-free survival analyses of NTRK1, COPS5, HIST1H3A, and HIST1H3G about LSCC. (e)–(g) Overall survival analyses of HIST1H3J, HIST1H3F, and ITGA4 about LSCC.

To demonstrate the relationship between key genes and LSCC, we analyzed the genes in core module through the GEPIA online database and Kaplan–Meier curve. We found that LSCC patients with high expression of HIST1H3J, HIST1H3F, and ITGA4 had worse overall survival, while LSCC patients with high expression of NTRK1, COPS5, HIST1H3A, and HIST1H3G had significantly worse disease-free survival (Figure 7).

## 4. Discussion

Based on the network pharmacology analysis of drug and disease target, collateral relationship can effectively reveal the mechanism of ZJC in the treatment of LSCC. Here, we found 96 candidate targets of ZJC and 81 DEGs of LSCC. Then, we constructed the PPI network for them separately. The huge genes involved in the interacted PPI network were analyzed to derive the possible mechanisms of anti-LSCC of ZJC, including transcriptional misregulation cancer, alcoholism, and cell cycle.

In our study, we identified 11 active ingredients of ZJC, which synergistically regulated 96 candidate targets. A large number of published literatures showed that the 11 active ingredients had anticancer activities, respectively. As reported, fisetin could inhibit the proliferation and migration of human laryngeal cancer via ERK1/2 and AKT/NF-KB/mTOR signaling pathways and induce apoptosis in human lung cancer through the MAPK signaling pathway [15, 16]. It was also revealed that kaempferol and quercetin were potential to inhibit cell migration and invasion in human head and neck squamous cell carcinoma [17, 18]. Li et al. emphasized that taxifolin may arrest aggressive breast cancer by promoting the MET progress through decreasing the expression of *β*-catenin [19]. Additionally, the inhibitory potency of flavanone on human breast cancer and gastric cancer has been reported previously [20, 21]. To our knowledge, no previous studies have explored the synergistic effect of the 11 active ingredients deriving from ZJC in suppressing LSCC development.

To investigate the possible mechanism of anti-LSCC of ZJC at a system level, we applied GlueGO to complete KEGG enrichment signaling pathway analysis, through analyzing the huge targets of the core PPI network in tight corresponding to LSCC and ZJC. We identified 11 items, in particular, transcriptional misregulation in cancer, alcoholism, cell cycle, and central carbon metabolism in cancer (all *P* < 0.01). It is apparent that both signal pathways of transcriptional misregulation in cancer and central carbon metabolism in cancer were closely associated with cancer [22, 23]. Sequentially, transcriptional misregulation in cancer was the most significant pathway following ZJC acting on LSCC (*P* < 0.001). As reported, cancer is more likely to occur in the mucous membrane in direct contact with alcohol; therefore, an intermediate increase in the risk of laryngeal cancer was found among alcoholics [24]. Aberrant cell cycle results in uncontrolled proliferation of cells, which is the common nature of cancer [25]. Zhou et al. demonstrated that Erchen decoction plus Huiyanzhuyu decoction was promising medicine in treatment of LSCC through inhibiting the cell cycle and inducing apoptosis of LSCC cells [26]. Protein processing in endoplasmic reticulum (ER) is crucial for the pathogenesis of cancer, with severe ER stress closely related to the development and invasion of cancer [27, 28]. These findings were consistent with the network pharmacology analysis.

## 5. Conclusion

Our study revealed that the anti-LSCC mechanism of ZJC was closely connected to transcriptional misregulation cancer, alcoholism, and cell cycle signaling pathway, which provided an important basis for further discussion of the follow-up experiment al design, making the experimental research more reasonable and more instructive.

## Figures and Tables

**Table 1:** The active ingredients in ZJC.

No.	Component	OB (%)	DL
1	Fisetin	52.60	0.24
2	Fustin	50.91	0.24
3	(-)-Taxifolin	60.51	0.27
4	Flavanone	41.35	0.24
5	Beta-sitosterol	36.91	0.75
6	Sitosterol	36.91	0.75
7	Kaempferol	41.88	0.24
8	Stigmasterol	43.83	0.76
9	Stigmast-4-ene-3,6-dione	39.12	0.79
10	Ent-epicatechin	48.96	0.24
11	Quercetin	46.43	0.28

**Table 2:** Differently expressed genes from GSE84957.

GeneSample	logFC	AveExpr	*t*	*P* value	Adj. *P* value	B
CST1	4.255518022	7.417759878	14.09776157	1.49*E* − 11	3.71*E* − 07	15.51198443
XLOC_004426	3.044228211	3.370726783	11.59258332	4.32*E* − 10	3.61*E* − 06	12.75595229
MMP11	3.903325333	6.986877144	11.58573097	4.36*E* − 10	3.61*E* − 06	12.74750381
GPRIN1	3.857745044	5.653573411	11.29565743	6.67*E* − 10	4.15*E* − 06	12.38486185
COL7A1	3.191737644	9.258242733	10.78042002	1.45*E* − 09	7.22*E* − 06	11.71597451
FAM3D	−5.792545067	7.2950313	−10.09792	4.24*E* − 09	1.51*E* − 05	10.77877059
LRP12	3.580821556	4.005304867	9.316160682	1.54*E* − 08	4.26*E* − 05	9.629111691
CTHRC1	4.740806433	7.128087172	9.196467428	1.89*E* − 08	4.30*E* − 05	9.445581444
LOC100506027	4.143287578	4.568671189	8.999347488	2.66*E* − 08	4.97*E* − 05	9.138857621
TJP3	−3.364634633	7.834813517	−8.904023211	3.14*E* − 08	4.97*E* − 05	8.9885142
IGFBP3	3.604138167	10.55244692	8.893742313	3.20*E* − 08	4.97*E* − 05	8.972220302
ARSI	3.152646867	3.986923189	8.298478812	9.26*E* − 08	0.000121313	8.002153147
TMEM158	3.718106033	4.697089117	8.222894187	1.06*E* − 07	0.000132353	7.875178695
PLAUR	3.027472033	7.824632761	8.051498015	1.46*E* − 07	0.000165132	7.584034636
MSR1	3.918547922	4.497970906	7.705672	2.80*E* − 07	0.000248682	6.98290287
TGFBI	3.415596633	9.235072794	7.534242043	3.89*E* − 07	0.000311941	6.678084284
SPP1	4.853097222	7.318721011	7.296283387	6.17*E* − 07	0.000415016	6.247440183
CYP4B1	−3.938406289	5.174530178	−7.145413133	8.31*E* − 07	0.000492716	5.96986977
SH3BGRL2	−4.071528089	6.438326422	−7.12999436	8.57*E* − 07	0.000495582	5.941304558
LOC100653149	3.268119356	5.443807811	7.078046014	9.50*E* − 07	0.000525005	5.844794163
MMP1	4.862459922	6.783056994	7.002232691	1.10*E* − 06	0.000590601	5.70320204
TM4SF19	4.052795678	5.81647465	6.87435807	1.43*E* − 06	0.000696569	5.462380919
COL5A2	3.504323078	7.584409283	6.759032897	1.80*E* − 06	0.000815742	5.243053839
HOXD11	3.550990856	5.087563172	6.54672159	2.78*E* − 06	0.001117349	4.834020589
CXCL11	4.762321211	4.652769394	6.519758265	2.94*E* − 06	0.00115171	4.781590785
LOC100652832	3.253860656	6.465006439	6.502526246	3.05*E* − 06	0.00115171	4.748026804
CXCL10	4.519192844	5.640273644	6.498769364	3.07*E* − 06	0.00115171	4.740703422
XLOC_006053	−3.635899633	9.156396294	−6.475756406	3.22*E* − 06	0.00115171	4.69579809
KRT17	4.404712111	11.51599561	6.464235069	3.30*E* − 06	0.00115171	4.673287016
PTHLH	4.098820033	7.949679894	6.341212465	4.26*E* − 06	0.001315184	4.431701824
CXCL12	−3.476515767	8.330393117	−6.24092991	5.26*E* − 06	0.001422596	4.233143024
XLOC_l2_006021	4.372459256	11.50269782	6.229963809	5.38*E* − 06	0.001424781	4.211342414
COL8A1	3.211121667	6.060922078	6.177430972	6.01*E* − 06	0.001526438	4.106669064
CLCA4	−5.919341656	8.217143061	−6.126082361	6.70*E* − 06	0.001661254	4.003977039
HMGA2	3.706329122	3.703429261	6.117033971	6.83*E* − 06	0.00166615	3.985842669
WISP1	3.021981556	5.595450789	6.039266244	8.06*E* − 06	0.001838778	3.829512486
NRG1	3.430506311	6.615773067	6.013403137	8.51*E* − 06	0.001907796	3.777336092
ANKRD20A9P	−3.027104311	6.964110233	−5.991259577	8.92*E* − 06	0.001947336	3.732590438
GCNT3	−3.483360222	5.808183467	−5.968076797	9.38*E* − 06	0.001965485	3.685672938
MYOC	−3.290936644	3.378123278	−5.964261662	9.45*E* − 06	0.001965485	3.677944815
ODZ2	3.030850211	7.342800339	5.955698768	9.63*E* − 06	0.001965485	3.660592194
DNAPTP3	4.162512589	8.355008817	5.948057503	9.79*E* − 06	0.001979224	3.645098841
FUT3	−3.452809133	7.136305	−5.832221646	1.25*E* − 05	0.00221345	3.409270286
CRNN	−6.524733278	9.72241585	−5.821941297	1.28*E* − 05	0.002227034	3.388254529
CFD	−3.520037456	9.369048272	−5.800510125	1.34*E* − 05	0.002286888	3.344398953
COL1A2	3.096446333	11.78684306	5.800402532	1.34*E* − 05	0.002286888	3.34417863
CXCL9	3.067348622	6.615663178	5.759709415	1.47*E* − 05	0.002369549	3.260741185
PDPN	3.170275733	10.0438188	5.709284988	1.64*E* − 05	0.00252911	3.157054467
INHBA	3.099887078	5.809753317	5.656043661	1.84*E* − 05	0.002640463	3.04722486
MMP12	4.162521167	3.736549417	5.638864584	1.91*E* − 05	0.002687547	3.011710913
SCARA5	−3.861003467	7.106386856	−5.634806253	1.92*E* − 05	0.002687547	3.003315853
COL4A1	3.293993722	11.03886292	5.623430739	1.97*E* − 05	0.002709232	2.979773593
COL5A1	3.205179622	8.674301744	5.604407653	2.05*E* − 05	0.00279295	2.940368581
ANKRD20A5P	−3.118826611	5.357065783	−5.574210889	2.19*E* − 05	0.002873117	2.877727019
TNXB	−3.390147544	8.386793894	−5.46437267	2.79*E* − 05	0.003345526	2.648949986
CCDC25	−3.340572133	7.218539933	−5.461095079	2.81*E* − 05	0.003345526	2.6421014
FN1	3.580535444	11.63254756	5.427958008	3.02*E* − 05	0.003525959	2.572791248
MAL	−6.360331133	8.9132381	−5.380498508	3.36*E* − 05	0.003726774	2.473306046
FBN2	3.702098767	4.647001128	5.317540391	3.86*E* − 05	0.003963698	2.340945176
KRT4	−5.783585056	12.44990214	−5.27501498	4.24*E* − 05	0.004304541	2.251297748
MSC	3.021481811	5.778950494	5.273063535	4.25*E* − 05	0.004304541	2.247179302
MYZAP	−3.966621	6.975986556	−5.218924365	4.80*E* − 05	0.00453758	2.132761995
CHI3L1	3.447755856	8.454925183	5.212706409	4.86*E* − 05	0.004565936	2.119601631
XLOC_008370	−4.086801056	5.071876417	−5.195774385	5.05*E* − 05	0.004618685	2.083744902
AMY1C	−3.066162922	4.171807861	−5.181124682	5.22*E* − 05	0.004702673	2.052698071
FAM107 A	−3.366929044	6.613174211	−5.153177492	5.55*E* − 05	0.004829406	1.993410827
CA9	4.004719489	4.832498267	5.105740988	6.17*E* − 05	0.005199505	1.89260426
SFI1	−3.172654489	7.898643289	−5.038975245	7.16*E* − 05	0.005657681	1.75036129
KRT24	−4.307497756	6.663276378	−4.903497209	9.71*E* − 05	0.006802832	1.460515802
FAM3B	−3.002864178	7.472917967	−4.851035631	0.000109236	0.007385034	1.347873623
PSCA	−4.424161267	6.177034822	−4.836697273	0.000112824	0.007525305	1.317050516
TREM1	3.132156567	5.329140306	4.802412753	0.000121894	0.007755997	1.243287728
MMP7	4.0077695	8.433879583	4.797447382	0.000123267	0.007808567	1.232597743
XLOC_l2_007931	−3.335940244	10.48608082	−4.789159173	0.000125595	0.007875182	1.214750103
KRT6B	4.076616322	9.703940506	4.773184049	0.000130209	0.007978995	1.180336064
SERPINE1	3.5038576	7.4466057	4.752919626	0.00013631	0.008152053	1.136656679
ABCA8	−3.2918408	5.542601178	−4.749840302	0.000137262	0.008169717	1.130016856
SNX31	−3.294362333	3.714450967	−4.727065435	0.000144517	0.008363625	1.080888859
KRT16	3.956891967	10.74006252	4.71119429	0.000149801	0.008567571	1.046633089
RSAD2	3.410952911	5.3110705	4.667343136	0.000165442	0.009086152	0.951904596
CEACAM5	−3.982757711	9.060092478	−4.649201732	0.000172387	0.009343839	0.912681384

**Table 3:** The topological properties of the 60 key nodes.

Name (the key targets)	Degree	Betweenness	Betweenness centrality	Closeness	Closeness centrality	Topological coefficient
YWHAZ	155	3001.377571	0.01183919	0.585365854	0.58536585	0.14373835
YWHAG	87	816.3912445	0.00322033	0.537313433	0.53731343	0.16424877
MCM5	101	1080.133008	0.00426068	0.542518837	0.54251884	0.14457089
STAU1	114	1407.485881	0.00555195	0.550218341	0.55021834	0.13468492
ITGA4	208	7021.25165	0.02769593	0.616891065	0.61689106	0.10712371
APP	125	5666.777349	0.02235309	0.557522124	0.55752212	0.10929128
CUL3	211	7900.538977	0.03116436	0.626865672	0.62686567	0.1083315
CUL2	121	2193.490854	0.00865241	0.556906077	0.55690608	0.1311238
CUL4A	84	1104.388869	0.00435636	0.519052523	0.51905252	0.14961657
CUL1	158	2737.270054	0.0107974	0.579976985	0.57997699	0.12774443
COPS5	173	4596.081368	0.01812964	0.592941176	0.59294118	0.11812865
YWHAQ	116	2340.92677	0.00923399	0.5532382	0.5532382	0.13955671
EEF1A1	111	2303.865101	0.0090878	0.553846154	0.55384615	0.15726594
OBSL1	151	2614.051749	0.01031135	0.572077185	0.57207719	0.11367624
TARDBP	99	902.4212542	0.00355968	0.540192926	0.54019293	0.14812918
HSPA8	102	1110.484051	0.0043804	0.549019608	0.54901961	0.1617004
HSPA5	124	2786.82878	0.01099289	0.56187291	0.56187291	0.15308876
HSP90AA1	115	3058.241745	0.0120635	0.558139535	0.55813953	0.13921995
EIF4A3	94	923.5252369	0.00364293	0.535031847	0.53503185	0.14405585
HSP90AB1	111	2024.466508	0.00798568	0.555066079	0.55506608	0.14751182
MYC	88	1227.898615	0.00484355	0.536741214	0.53674121	0.11599255
CCDC8	145	2628.810161	0.01036957	0.568848758	0.56884876	0.11392955
HDAC1	84	1756.679856	0.00692938	0.525547445	0.52554745	0.11154264
RPA1	106	1443.910092	0.00569563	0.544864865	0.54486486	0.13668578
RPA2	98	1508.905701	0.00595201	0.541353383	0.54135338	0.13008096
HNRNPA1	130	1803.567503	0.00711433	0.565656566	0.56565657	0.14709052
EGFR	143	4724.066642	0.01863449	0.572727273	0.57272727	0.1103327
HIST1H3F	86	437.5273205	0.00172586	0.529411765	0.52941176	0.14705882
UBC	133	3538.655204	0.01395853	0.566929134	0.56692913	0.12972445
CUL7	174	4304.231567	0.01697841	0.594339623	0.59433962	0.10695547
HNRNPK	90	914.3729176	0.00360682	0.535600425	0.53560043	0.17748918
TUBB	85	1017.885462	0.00401514	0.537886873	0.53788687	0.16372149
HNRNPU	106	2391.698718	0.00943426	0.547826087	0.54782609	0.15722622
CDK2	172	5391.386868	0.02126679	0.592941176	0.59294118	0.11382953
EWSR1	85	1514.316862	0.00597335	0.532206969	0.53220697	0.15183716
CDC5L	113	2162.954989	0.00853196	0.550819672	0.55081967	0.13024984
NEDD8	86	533.4516789	0.00210425	0.528301887	0.52830189	0.14769424
HIST1H3A	86	437.5273205	0.00172586	0.529411765	0.52941176	0.14705882
HIST1H3D	86	437.5273205	0.00172586	0.529411765	0.52941176	0.14705882
TRAF6	100	2757.045931	0.01087541	0.538461538	0.53846154	0.09652163
HIST1H3C	86	437.5273205	0.00172586	0.529411765	0.52941176	0.14705882
NPM1	156	3506.462925	0.01383155	0.58400927	0.58400927	0.14115178
HIST1H3E	86	437.5273205	0.00172586	0.529411765	0.52941176	0.14705882
HIST1H3I	86	437.5273205	0.00172586	0.529411765	0.52941176	0.14705882
HIST1H3G	86	437.5273205	0.00172586	0.529411765	0.52941176	0.14705882
FUS	109	1065.023899	0.00420108	0.551422319	0.55142232	0.157963
HIST1H3J	86	437.5273205	0.00172586	0.529411765	0.52941176	0.14705882
HIST1H3H	86	437.5273205	0.00172586	0.529411765	0.52941176	0.14705882
HIST1H3B	86	437.5273205	0.00172586	0.529411765	0.52941176	0.14705882
NTRK1	270	16394.02825	0.06466766	0.673796791	0.67379679	0.09989618
FLNA	89	1820.058448	0.00717938	0.537313433	0.53731343	0.15216314
FN1	307	27515.32665	0.10853658	0.708860759	0.70886076	0.09048518
SIRT7	128	2444.801055	0.00964373	0.5532382	0.5532382	0.11177503
GRB2	136	4533.847683	0.01788415	0.570135747	0.57013575	0.12538522
FBXO6	129	2240.28371	0.00883699	0.558139535	0.55813953	0.11844315
VCP	103	1651.524516	0.00651458	0.544864865	0.54486486	0.14998166
CAND1	152	2355.429527	0.0092912	0.578645235	0.57864524	0.12845528
XPO1	106	2782.081388	0.01097416	0.543103448	0.54310345	0.12465874
MCM2	207	6633.522707	0.0261665	0.621454994	0.62145499	0.11887293
YWHAE	93	1174.476977	0.00463283	0.537313433	0.53731343	0.16260551

## Data Availability

The original data series GSE84957 used to support the findings of this study is downloaded from the Gene Expression Omnibus (GEO) microarray dataset.

## References

[1] Pukander H. R. J., Pukander J. (2009). Symptoms of laryngeal carcinoma and their prognostic significance. *Acta Oncologica*.

[2] Thompson L. D. R., Lester D. R. (2017). Laryngeal dysplasia, squamous cell carcinoma, and variants. *Surgical Pathology Clinics*.

[3] Hunter K. D., Parkinson E. K., Harrison P. R. (2005). Profiling early head and neck cancer. *Nature Reviews Cancer*.

[4] Zhu J., Fedewa S., Chen A. Y. (2012). The impact of comorbidity on treatment (chemoradiation and laryngectomy) of advanced, nondistant metastatic laryngeal cancer. *Archives of Otolaryngology-Head & Neck Surgery*.

[5] Zhang J.-P., Tian X.-H., Yang Y.-X. (2016). Gleditsia species: an ethnomedical, phytochemical and pharmacological review. *Journal of Ethnopharmacology*.

[6] Moon S. J., Cho Y. H., Kim H. (2009). Inhibitory effects of the ethanol extract of Gleditsia sinensis thorns on human colon cancer HCT116 cells in vitro and in vivo. *Oncology Reports*.

[7] Chow L. M. C., Tang J. C. O., Teo I. T. N. (2002). Antiproliferative activity of the extract of Gleditsia sinensis fruit on human solid tumour cell lines. *Chemotherapy*.

[8] Tang K. C., Lam K. Y., Law S. (1998). The inhibitory effect of Gleditsia sinensis on cyclooxygenase-2 expression in human esophageal squamous cell carcinoma. *International Journal of Molecular Medicine*.

[9] Liang X., Li H., Li S. (2014). A novel network pharmacology approach to analyse traditional herbal formulae: the Liu-Wei-Di-Huang pill as a case study. *Molecular BioSystems*.

[10] Wang N., Zheng Y., Gu J. (2017). Network-pharmacology-based validation of TAMS/CXCL-1 as key mediator of XIAOPI formula preventing breast cancer development and metastasis. *Scientific Reports*.

[11] Lee A. Y., Park W., Kang T.-W., Cha M. H., Chun J. M. (2018). Network pharmacology-based prediction of active compounds and molecular targets in Yijin-tang acting on hyperlipidaemia and atherosclerosis. *Journal of Ethnopharmacology*.

[12] Liu X., Ouyang S., Yu B. (2010). PharmMapper server: a web server for potential drug target identification using pharmacophore mapping approach. *Nucleic Acids Research*.

[13] Martin A., Ochagavia M. E., Rabasa L. C., Miranda J., Fernandez-de-Cossio J., Bringas R. (2010). BisoGenet: a new tool for gene network building, visualization and analysis. *Bmc Bioinformatics*.

[14] Tang Z., Li C., Kang B., Gao G., Li C., Zhang Z. (2017). GEPIA: a web server for cancer and normal gene expression profiling and interactive analyses. *Nucleic Acids Research*.

[15] Zhang X.-J., Jia S.-S. (2016). Fisetin inhibits laryngeal carcinoma through regulation of AKT/NF-*κ*B/mTOR and ERK1/2 signaling pathways. *Biomedicine & Pharmacotherapy*.

[16] Klimaszewska-Wisniewska A., Halas-Wisniewska M., Tadrowski T., Gagat M., Grzanka D., Grzanka A. (2016). Paclitaxel and the dietary flavonoid fisetin: a synergistic combination that induces mitotic catastrophe and autophagic cell death in A549 non-small cell lung cancer cells. *Cancer Cell International*.

[17] Chan C.-Y., Lien C.-H., Lee M.-F., Huang C.-Y. (2016). Quercetin suppresses cellular migration and invasion in human head and neck squamous cell carcinoma (HNSCC). *Biomedicine*.

[18] Swanson H. I., Choi E.-Y., Helton W. B., Gairola C. G., Valentino J. (2014). Impact of apigenin and kaempferol on human head and neck squamous cell carcinoma. *Oral Surgery, Oral Medicine, Oral Pathology and Oral Radiology*.

[19] Li J., Hu L., Zhou T. (2019). Taxifolin inhibits breast cancer cells proliferation, migration and invasion by promoting mesenchymal to epithelial transition via *β*-catenin signaling. *Life Sciences*.

[20] Park C. H., Hahm E. R., Lee J. H., Jung K. C., Yang C. H. (2005). Inhibition of *β*-catenin-mediated transactivation by flavanone in AGS gastric cancer cells. *Biochemical and Biophysical Research Communications*.

[21] Kim E., Lee J., Kim G. (2009). Anti-carcinogenic effect of a new analogue 4’-chloroflavanone from flavanone in human breast cancer cells. *International Journal of Molecular Medicine*.

[22] Lee T. I., Young R. A. (2013). Transcriptional regulation and its misregulation in disease. *Cell*.

[23] Wong T. L., Che N., Ma S. (2017). Reprogramming of central carbon metabolism in cancer stem cells. *Biochimica Et Biophysica Acta Molecular Basis of Disease*.

[24] McMichael A. J. (1978). Increases in laryngeal cancer in Britain and Australia in relation to alcohol and tobacco consumption trends. *Lancet*.

[25] Otto T., Piotr S. (2017). Cell cycle proteins as promising targets in cancer therapy. *Nature Reviews Cancer*.

[26] Zhou S. Q., Luo Q. L., Tan X. (2020). Erchen decoction plus huiyanzhuyu decoction inhibits the cell cycle, migration and invasion and induces the apoptosis of laryngeal squamous cell carcinoma cells. *Journal of Ethnopharmacology*.

[27] Moon H. W., Han H. G., Jeon Y. J. (2018). Protein quality control in the endoplasmic reticulum and cancer. *International Journal of Molecular Sciences*.

[28] Mohamed E., Cao Y., Rodriguez P. C. (2017). Endoplasmic reticulum stress regulates tumor growth and anti-tumor immunity: a promising opportunity for cancer immunotherapy. *Cancer Immunology Immunotherapy Cii*.

